# Anti-Fibrotic Effects of Low Toxic Microcystin-RR on Bleomycin-Induced Pulmonary Fibrosis: A Comparison with Microcystin-LR

**DOI:** 10.3389/fphar.2021.675907

**Published:** 2021-06-08

**Authors:** Jie Wang, Yan Ren, Xiufen Zheng, Jiaqi Kang, Zhenqian Huang, Lizhi Xu, Yaping Wang

**Affiliations:** ^1^ Department of Medical Genetics, Nanjing University School of Medicine, Nanjing, China; ^2^ Department of Tumor Biobank, Jiangsu Cancer Hospital, Jiangsu Institute of Cancer Research, Nanjing Medical University Affiliated Cancer Hospital, Nanjing, China; ^3^ Jiangsu Key Laboratory of Molecular Medicine, Nanjing University School of Medicine, Nanjing, China; ^4^ Department of Pharmacy, The First Affiliated Hospital of Hainan Medical University, Haikou, China

**Keywords:** pulmonary fibrosis, microcystin-RR, microcystin-LR, macrophages, bleomycin

## Abstract

Idiopathic pulmonary fibrosis (IPF) is a chronic progressive interstitial pulmonary disease characterized with radiographically evident pulmonary infiltrates and extracellular matrix deposition with limited treatment options. We previously described that microcystin-LR (MC-LR) reduces transforming growth factor (TGF)-β1/Smad signaling and ameliorates pulmonary fibrosis in bleomycin (BLM)-induced rat models. In the present study, we further demonstrate that microcystin-RR (MC-RR), an MC congener with lower toxicity than MC-LR, exerted an anti-fibrotic effect on BLM-induced pulmonary fibrosis rodent models and compared it with MC-LR. Our data show that MC-RR treatment attenuated BLM-associated pulmonary inflammation and collagen deposition in both therapeutic and preventive models. MC-RR reduced the expression of fibrotic markers, including vimentin, α-smooth muscle actin, collagen 1α1, and fibronectin, in rat pulmonary tissues. Furthermore, the core features of BLM-induced pulmonary fibrotic lesions were better alleviated by MC-RR than by MC-LR. MC-RR treatment substantially decreased the number of pulmonary M2 macrophages. *In vitro*, MC-RR attenuated the epithelial-mesenchymal transition and fibroblast-myofibroblast transition triggered by M2 macrophages. Therefore, we highlight MC-RR as a promising molecule for developing therapeutic and prophylactic strategies against IPF, a refractory lung disease.

## Introduction

Idiopathic pulmonary fibrosis (IPF) is a progressive fibrotic lung disease characterized with inflammation, fibroblast accumulation, and collagen deposition (Lederer and Martinez, 2018; Sgalla et al., 2018; Chanda et al., 2019). Excessive fibrosis destroys the lung architecture, consequently leading to respiratory dysfunction and failure. IPF is an important lethal respiratory disease and represents a global challenge, with an incidence of 2–30 cases per 100,000 person-years worldwide and a median survival of 3–5 years after diagnosis. Two medications, nintedanib and pirfenidone, have received the Food and Drug Administration (FDA) approval for the treatment of IPF and are mainly used to decelerate the rate of decline in forced vital capacity (Martinez and Lederer, 2018; Collins and Raghu, 2019; Somogyi et al., 2019). However, neither of them has shown survival benefits with reduced mortality, highlighting the urgent need for development of novel therapies for IPF.

Evidence supports the role of macrophages in the pathogenesis of pulmonary flbrosis. Macrophages have remarkable plasticity and can shift from one functional phenotype to another (Wynn et al., 2013; Wynn and Vannella, 2016; Locati et al., 2020; Ogger and Byrne, 2020). Two distinct populations of macrophages are widely described in pulmonary fibrosis pathogenesis, namely, classically activated (M1) macrophages and alternatively activated (M2) macrophages. An expanded population of M2 macrophages is believed to be involved in the development and maintenance of IPF. M2 macrophages migrate to the injury sites and act as vital regulators of fibrogenesis by inducing myofibroblast differentiation and excessive collagen production. The interplay between M1 and M2 macrophages influences the outcome and severity of IPF. Therefore, strategies aimed at modulating lung macrophages are considered promising for the prevention and treatment of IPF (Morse et al., 2019; Ucero et al., 2019; Pan et al., 2021).

Microcystins (MCs) produced by cyanobacteria have more than 100 different congeners that share the basic structure of monocyclic heptapeptides but exhibit highly variable toxicity. A single amino acid difference in its molecular structure deems microcystin-LR (MC-LR) as a relatively toxic congener, as its acute or subchronic exposure has been shown to cause health damage, especially hepato-, nephro-, and neuro-toxicity. Microcystin-RR (MC-RR), on the contrary, has decreased toxicity (Gupta et al., 2003; Wu et al., 2017; Wang et al., 2018; Kordasht et al., 2020; Zhao et al., 2020). The primary toxicologic mechanism of MCs has been well established and related to the inhibition of the catalytic subunit of protein phosphatases 1 and 2A (PP1 and PP2A) that perform hyperphosphorylation of cellular proteins and are associated with signaling events related to the cytoskeleton and cell metabolism, proliferation, and apoptosis (Campos and Vasconcelos, 2010; Sassolas et al., 2011; Liu and Sun, 2015; Fontanillo et al., 2016; Fontanillo and Köhn, 2018).

We have previously described that MC-LR exhibits therapeutic potential for pulmonary fibrosis (Wang et al., 2020) by demonstrating its ability to attenuate the macrophage polarization toward the CD206^+^ M2-like phenotype. This effect results in the inhibition of epithelial-mesenchymal transition (EMT) and fibroblast-myofibroblast transition (FMT) signaling in epithelial cells and fibroblasts. MC-LR interacts with glucose-regulated protein 78 kDa, a master regulator of endoplasmic reticulum stress, and blocks the endoplasmic reticulum unfolded protein response signal transduction in stressed cells. The attenuation of M2 macrophages results in the downregulation of transforming growth factor-β1 (TGF-β1) expression and TGF-β1/Smad signaling, thus contributing to the amelioration of pulmonary fibrosis. Given the potential biological toxicity of MC-LR, we selected MC-RR, a low toxic congener of MCs, and assessed its preventive and therapeutic effects in BLM-induced pulmonary fibrosis. Further, we compared MC-RR with MC-LR. Our data confirm that both therapeutic and preventive MC-RR regimens are effective in alleviating pulmonary fibrosis in rodent models, highlighting its translational value.

## Materials and Methods

### Chemicals

MC-RR and MC-LR were purchased from Alexis Biochemicals (Lausen, Switzerland), and BLM was obtained from Nippon Kayaku Co. (Tokyo, Japan). Murine interleukin (IL)-4 was supplied by PeproTech (Rocky Hill, NJ, United States).

### Therapeutic Model

Six- to eight-week-old male SD rats (220–250 g) were intratracheally instilled with a single dose of BLM (5.0 mg/kg) to induce pulmonary fibrosis. Some rats received MC-RR (20 μg/L) in drinking water from day 14–56 following BLM instillation. Control rats received an equal volume of sterile saline and distilled water daily. The rats were euthanized 56 days after receiving BLM intratracheal instillation for further analysis.

### Preventive Model

Six- to eight-week-old male C57BL/6 mice (20–22 g) were induced using a single intratracheal instillation of BLM (3 mg/kg). Some mice received MC-RR (20 μg/L) in drinking water 7 days before BLM administration until 28 days after BLM induction. The control mice were subjected to the same procedure but received intratracheal instillation of saline and distilled water. All mice underwent a computerized tomography (CT) scan and were euthanized on day 28 after BLM instillation. The pulmonary tissues were collected for histopathological observation.

### Comparative Model

Male SD rats (220–250 g) aged 6–8 weeks received a single intratracheal instillation of 5.0 mg/kg BLM or equal volume of saline on day 0. Some rats with fibrosis received MC-RR or MC-LR (20 μg/L) in drinking water, starting on day 7 (RR7 or LR7), 14 (RR14 or LR14), and 28 (RR28 or LR28) until day 56 after BLM treatment.

### Micro-CT Imaging

Mice were anesthetized with isoflurane 2% (vol/vol) in O_2_ and transferred into a high-resolution micro-CT (SkyScan 1176, Bruker) to scan the chest area.

### Hydroxyproline (HYP) Assay

The HYP content of the lungs from model animals was determined using an HYP assay kit (Jiancheng Bioengineering Institute, Nanjing, China) according to the manufacturer’s instructions. The absorbance of each sample was determined at 550 nm wavelength using a SpectraMax M2e microplate reader (Molecular Devices, Sunnyvale, CA, United States). The amount of collagen was expressed in micrograms per milligram of the lung tissue.

### Histology and Immunostaining

Paraffin-embedded tissue sections were deparaffinized, rehydrated, and rinsed with distilled water. The samples were subjected to hematoxylin and eosin (H&E), Masson’s trichrome, Sirius Red, or Elastica-van Gieson (EVG) staining. Some sections of pulmonary tissues were also subjected to antigen retrieval in boiling 0.1 M citrate (PH 6.0) buffer for 10 min and then probed with antibodies against vimentin (5147S, CST, Beverly, Massachusetts, United States), collagen 1α1 (ab138492, Abcam, Cambridge, Massachusetts, United States), CD206 (ab64693, Abcam), and inducible nitric oxide synthase (iNOS, ab178945, Abcam). Histological images were visualized using a Zeiss Axio microscope.

### Cell Culture

Human (L02 and A549) or mouse (NIH3T3 and RAW264.7) cell lines were purchased from the Cell Bank of the Typical Culture Preservation Commission, Chinese Academy of Sciences, and cultured in Dulbecco’s modified Eagle’s medium (DMEM) or Roswell Park Memorial Institute (RPMI)-1640 medium supplemented with 10% fetal bovine serum (Thermo Fisher Scientific). All cells were confirmed negative for *mycoplasma* contamination using a MycoBlue™ *mycoplasma* detector (Vazyme, Nanjing, China).

### Treatment of Cultured Cells and Co-Culture

RAW264.7 cells seeded at a density of 1 × 10^5^ cells/ml were exposed to IL-4 (5 ng/ml) for 48 h to induce M2-like differentiation. Some cells were treated with 0.1 μM MC-LR or MC-RR. To establish a co-culture with M2-like macrophages, we transferred cell culture inserts containing IL-4-pretreated macrophages to plates seeded with A549 or NIH3T3 cells (5 × 10^4^ cells/ml) for 24 h. After 48 h of co-culture, A549 or NIH3T3 cells at the bottom of plates were harvested for further experiments.

### Cell Proliferation Assay

The cell yield was monitored in real time using the xCELLigence system (ACEA Biosciences Inc., San Diego, CA, United States). First, impedance baseline measurements were obtained for wells of an E-Plate containing 50 μl medium. Subsequently, 5,000 cells were seeded in fresh medium at a final volume of 150 μl. The E-Plate was incubated at 37°C with 5% CO_2_ for 16 h. MC-RR or MC-LR was added to the wells at indicated concentrations, and the impedance signals were recorded every 15 min until the end of the experiment.

### PP2A Phosphatase Activity Assay

PP2A activity was determined using a Serine/Threonine phosphatase Assay Kit according to the manufacturer’s instructions (Promega, Madison, Wisconsin, United States). Briefly, L02 cell lysates in phosphatase storage buffer were centrifuged and subsequently added to a reaction buffer for 1 h at 37°C. The reaction was stopped and quantified at 630 nm using a SpectraMax M2e microplate reader.

### Flow Cytometry

RAW264.7 cells were left alone or cultured with IL-4 (5 ng/ml) for 48 h. Some cells were also treated with 0.1 μM MC-LR or MC-RR. The cells were harvested, washed, and resuspended in 0.5% bovine serum albumin (BSA)-phosphate-buffered saline (PBS). Next, the cells were preincubated with mouse Fc block (eBioscience, San Diego, California, United States) for 15 min and subsequently stained with an Alexa Fluor 488-conjugated anti-CD11b Ab (eBioscience) and an allophycocyanin (APC)-conjugated anti-CD206 Ab (eBioscience) for 30 min at 4°C in the dark. Flow cytometric analyses were performed using a FACSCalibur flow cytometer (BD Biosciences, San Jose, CA, United States) and FlowJo software. Apoptotic cells were analyzed by flow cytometry using annexin V-fluorescein isothiocyanate (FITC) and propidium iodide (PI) staining (BD Biosciences). Some cells were fixed in 70% ethanol for overnight, stained with 10 μg/ml PI, and analyzed for cell cycle distribution by flow cytometry.

### Western Blot Analysis

Cells and tissues were washed and lysed in radioimmunoprecipitation assay (RIPA) buffer supplemented with protease and phosphatase inhibitors. Ten microliters of lysates were separated by 10% sodium dodecyl sulfate polyacrylamide gel electrophoresis (SDS-PAGE), and proteins were transferred onto Polyvinylidene fluoride (PVDF) membranes. Membranes were blocked in 5% nonfat dry milk and incubated with diluted primary antibodies at 4°C for overnight. The primary antibodies used were as follows: TGF-β1 (ab215715, Abcam), α-smooth muscle actin (αSMA; ab32575, Abcam), fibronectin (ab32419, Abcam), Smad2/3 (3102S, CST), p-Smad3 (9520S, CST), E-cadherin (3195S, Cell Signaling Technology), vimentin (5147S, CST), CD206 (ab125028, Abcam), and iNOS (ab178945, Abcam). All antibodies were 1:1,000 diluted in the blocking solution. Next, the PVDF membranes were washed thrice in TBS-T (10 mM Tris-HCl, pH 7.4; 150 mM NaCl; 0.1% Tween-20), incubated with an IRDye 800CW goat anti-mouse or IRDye 680CW goat anti-rabbit (Li-Cor Biosciences, Lincoln, Nebraska, United States) secondary antibody at 1:10,000 dilution in TBS-T, and washed. The signals were detected using an Odyssey scanner (Li-Cor Biosciences).

### Reverse-Transcription Quantitative Polymerase Chain Reaction Analysis

Total RNA isolated from cells and pulmonary tissues using TRIzol reagent (Thermo Fisher Scientific) was reverse transcribed using PrimeScript RT reagent kit (Takara, Shiga, Japan) and assayed for gene expression using Fast SYBR® Green Master Mix (Thermo Fisher Scientific) on a QuantStudio 6 (Applied Biosystems, Foster City, California, United States). Each sample was tested in triplicates, and at least two biological replicates were maintained in each assay. Glyceraldehyde 3-phosphate dehydrogenase (*GAPDH*) was used as an internal control, and all data were calculated using the ΔΔ cycle threshold (Ct) method. Gene-specific forward and reverse primer sequences are listed in Supplementary Table S1.

### Immunofluorescence Assay

Cells were washed twice in ice-cold PBS without calcium or magnesium, fixed in 4% paraformaldehyde (Thermo Fisher Scientific), and permeabilized with 0.1% Triton X-100 (Thermo Fisher Scientific). Cells were then incubated in 3% BSA (Sigma-Aldrich) to block cross-reactivity, and probed with specific primary antibodies for overnight at 4°C. The following primary antibodies were used at the indicated dilutions: MC-LR (Alexis Biochemicals, 1:200), vimentin (CST, 1:200), E-cadherin (CST, 1:200), fibronectin (Abcam, 1:200), and αSMA (Abcam, 1:200). The next day, cells were washed and stained with Alexa Fluor 488- and Alexa Fluor 549-labeled secondary antibodies (Thermo Fisher Scientific, 1:300) for 1 h at room temperature in the dark. Nuclear staining was performed with 4′,6-diamidino-2-phenylindole (DAPI; 1 μg/ml in PBS, Thermo Fisher Scientific) for 10 min at room temperature. Cells were washed, mounted onto glass slides, and visualized using an Olympus confocal laser scanning microscope imaging system and a Zeiss fluorescent microscope.

### Hematological Analysis and Serum Biochemistry

Blood samples were collected from the rats and evaluated using an auto-dry chemistry analyzer (Kehua Bioengineering, Shanghai, China) for serum parameters, including aspartate aminotransferase (AST), alanine aminotransferase (ALT), blood urea nitrogen (BUN), and creatinine (CRE), and the lipid profile.

### Statistical Evaluation

Statistical analysis was performed using SPSS (version 24.0; SPSS Inc., Chicago, Illinois, United States). Data are expressed as the mean ± standard deviation (SD). Statistical comparisons were performed using one-way analysis of variance (ANOVA) with Student-Newman-Keuls post-hoc analysis. Statistical significance was set at *p* < 0.05.

## Results

### MC-RR Markedly Attenuates BLM-Induced Pulmonary Fibrosis

**FIGURE 1:**
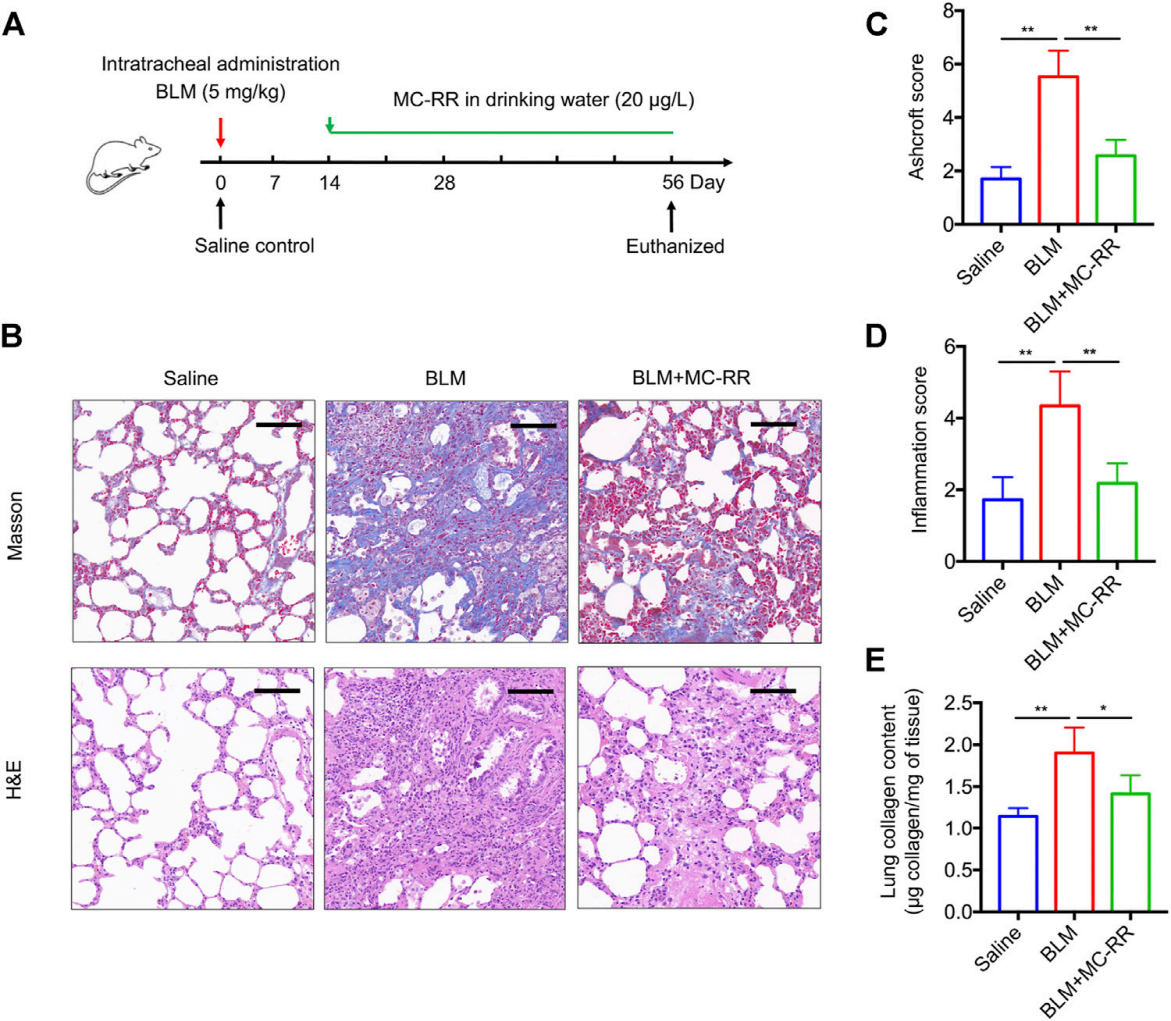
Microcystin-RR (MC-RR) attenuates bleomycin (BLM)-induced pulmonary fibrosis. Rats received a single intratracheal instillation of 5.0 mg/kg BLM on day 0. Some of these animals were treated with MC-RR (20 μg/L) in drinking water on day 14. The control rats received saline intratracheal administration and had distilled water for daily drinking. Rats were euthanized on day 56 after BLM instillation, and lung tissue samples were collected for analysis. **(A)** Schematic representation of the experimental design. **(B)** Lung tissue sections were prepared and subjected to Masson’s trichrome and H&E staining. Scale bar: 100 μm. **(C,D)** Ashcroft and inflammation scores of stained sections of lung tissues were assessed by two pathologists blinded to the study design and are shown as the mean ± SD per group. **(E)** Lung tissue collagen contents were measured by a hydroxyproline assay. Data are expressed as mean ± SD. *p < 0.05, **p < 0.01 determined by one-way ANOVA with S-N-K post-hoc analysis. Each group had five rats.

To investigate whether MC-RR exerts an anti-fibrotic effect, we established a BLM-induced rat model that was administered MC-RR from day 14–56 after BLM intratracheal instillation (Figure 1A). As expected, administration of BLM drove a series of IPF-associated morphological characteristics as compared with the control treatment. MC-RR treatment markedly ameliorated the pathological progression of fibrosis, as evident from the decreased phagocytic infiltration, reduced alveolar injury, less interstitial thickening, and mild accumulation of the extracellular matrix (Figure 1B). The pathology scores for fibrosis and inflammation of the lung tissue in MC-RR-treated rats were significantly lower than those in BLM model rats (Figures 1C,D). Consistent with histology results, MC-RR notably reduced the content of HYP, a representative marker for collagen deposition, in the lung tissue that was highly elevated in BLM model rats (Figure 1E). These data indicate that MC-RR exerts a therapeutic effect on BLM-induced pulmonary fibrosis in rats.

### MC-RR Exerts a Preventive Effect on Pulmonary Fibrosis Models

**FIGURE 2:**
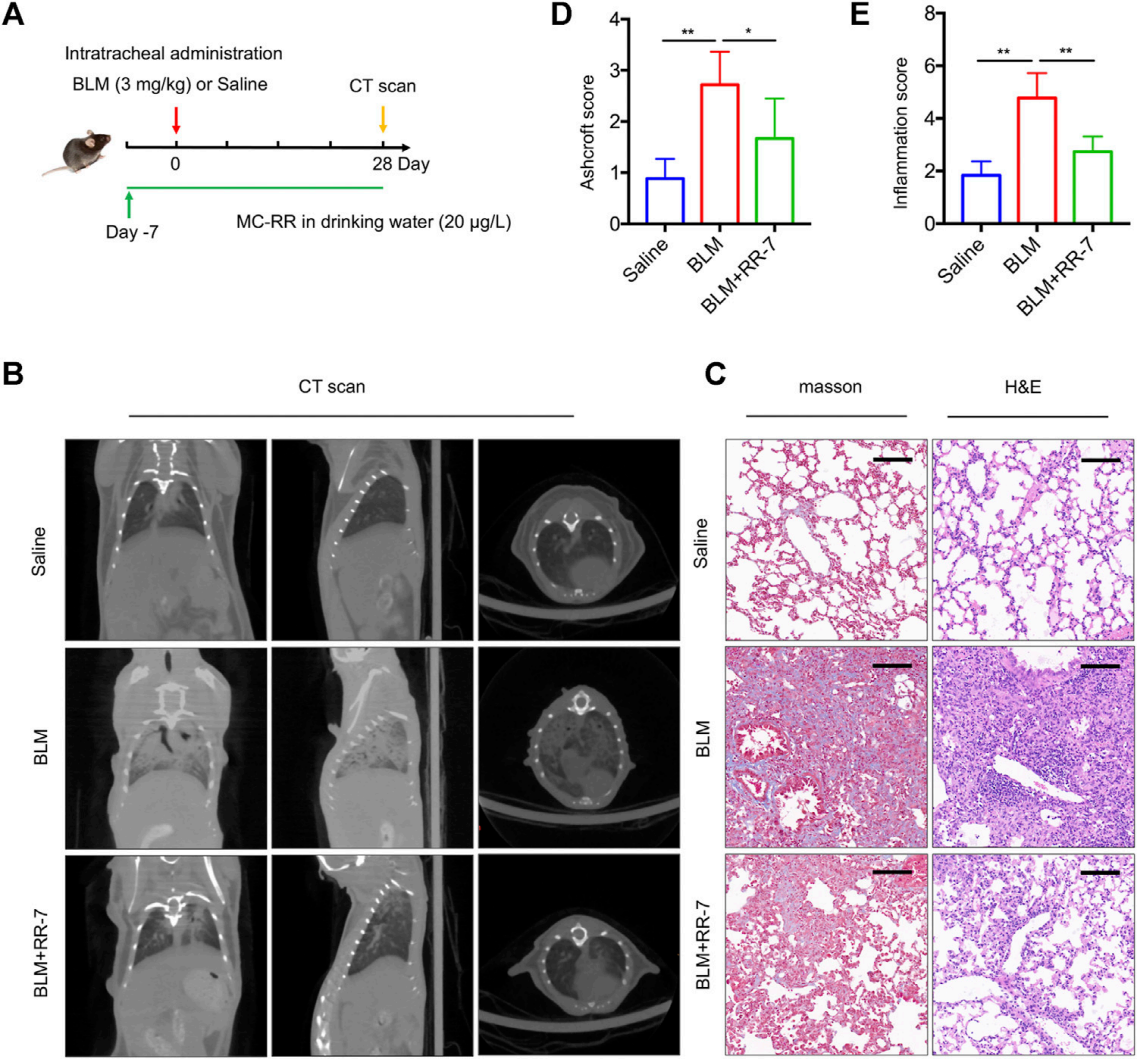
Microcystin-RR (MC-RR) exerts a preventive effect on pulmonary fibrosis models. Mice were treated with MC-RR (20 μg/L) in drinking water for 7 days and subsequently received intratracheal instillation with a single dose of bleomycin (BLM, 3.0 mg/kg), followed by MC-RR administration in drinking water for additional 28 days. The BLM group received distilled water instead of MC-RR. Mice that received intratracheal instillation of an equal volume of saline were used as control. On day 28 after BLM instillation, all mice were scanned with micro-CT and euthanized for analysis. **(A)** Schematic representation of the experimental design. **(B)** The radiologic characteristics of the lungs of mice were determined by Micro-CT. **(C)** Lung tissue sections were prepared and subjected to Masson’s trichrome and H&E staining. Scale bar: 100 μm. **(D,E)** Ashcroft and inflammation scores of stained sections of lung tissues were measured and are shown as the mean ± SD. Data are expressed as mean ± SD. *p < 0.05, **p < 0.01 determined by one-way ANOVA with S-N-K post-hoc analysis. Each group had five mice.

To explore the effect of MC-RR on the prevention of pulmonary fibrosis, we exposed mice to drinking water containing MC-RR 7 days prior to BLM intratracheal instillation. We assessed the preventive effect of MC-RR on pulmonary fibrosis until day 28 after BLM instillation (Figure 2A). As illustrated by the micro-CT of the mouse chest, the saline control group showed a normal lung tissue structure. However, BLM-exposed mice displayed the development of pulmonary parenchyma lesions with ground-glass opacities and honeycombing. The administration of MC-RR resulted in mild reticulation and distortion (Figure 2B), indicating the marked reduction in the lung lesions. Similarly, the preventive administration of MC-RR significantly attenuated the remodeling of the lung architecture and collagen fiber deposition, as evident from the changes in Ashcroft and inflammation scores in Masson and H&E staining (Figures 2C–E). These results suggest that the preventive regimen of MC-RR also mediates anti-fibrotic effects.

### MC-RR Alleviates Pulmonary Fibrosis Better than MC-LR

**FIGURE 3:**
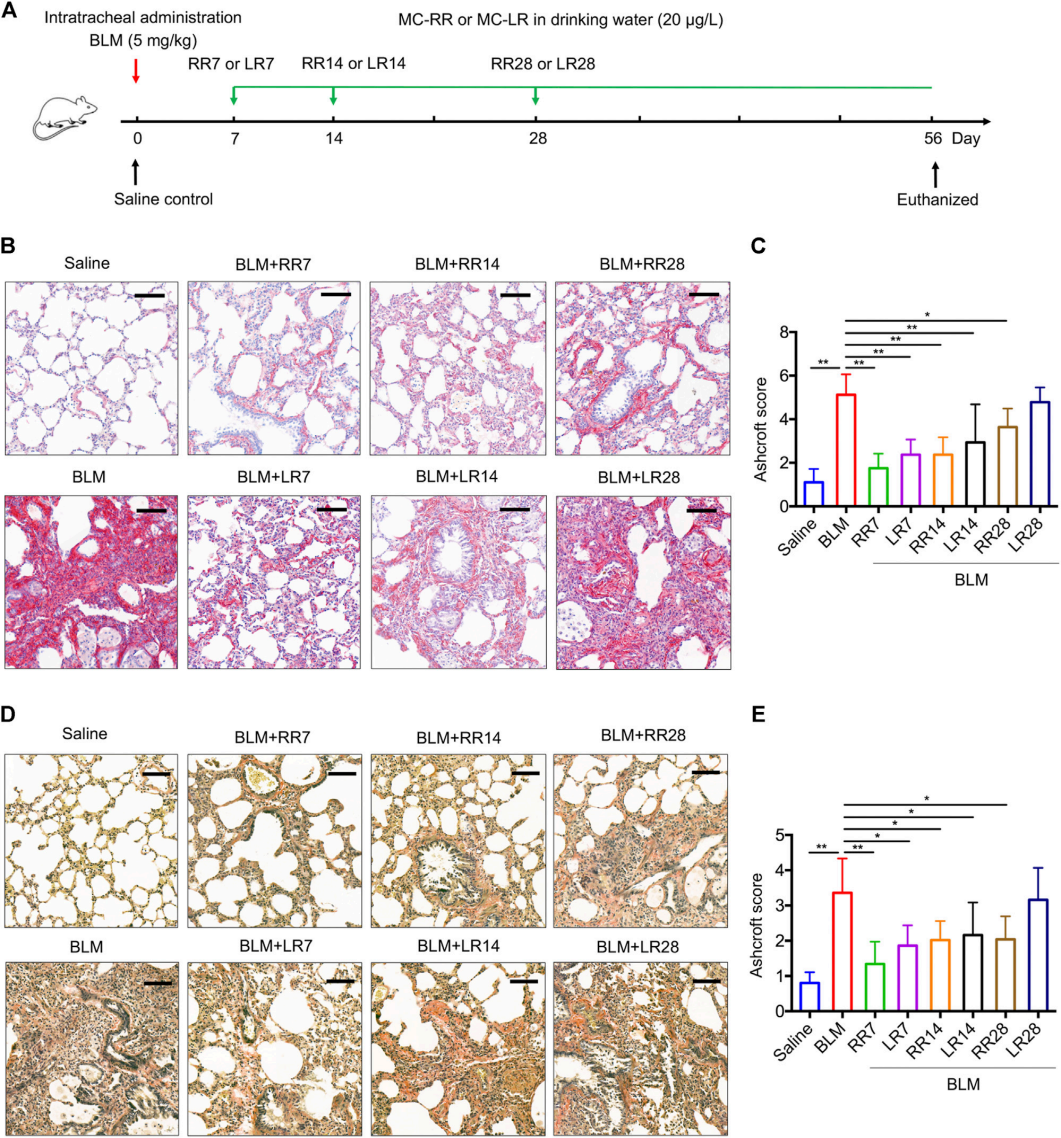
Microcystin-RR (MC-RR) inhibits deposition of collagen and elastic fibers better than Microcystin-LR (MC-LR). Rats were intratracheally instilled with a single dose of bleomycin (BLM, 5.0 mg/kg) on day 0 and started to receive MC-RR or MC-LR (20 μg/L) in drinking water on day 7 (LR7/RR7), 14 (LR14/RR14), or 28 (LR28/RR28). On day 56, rats were killed and their lung tissues were collected for analysis. **(A)** Schematic representation of the experimental design. **(B,C)** Lung tissue sections were prepared and subjected to Sirius Red staining. Scale bar: 100 μm. Ashcroft score of stained sections was shown as mean ± SD per group. **(D,E)** Elastic fibers of rat lung tissues were evaluated by Van Gieson staining. Scale bar: 100 μm. Ashcroft score of stained sections was presented as mean ± SD. *p < 0.05, **p < 0.01 determined by one-way ANOVA with S-N-K post-hoc analysis. Each group had five rats.

**FIGURE 4:**
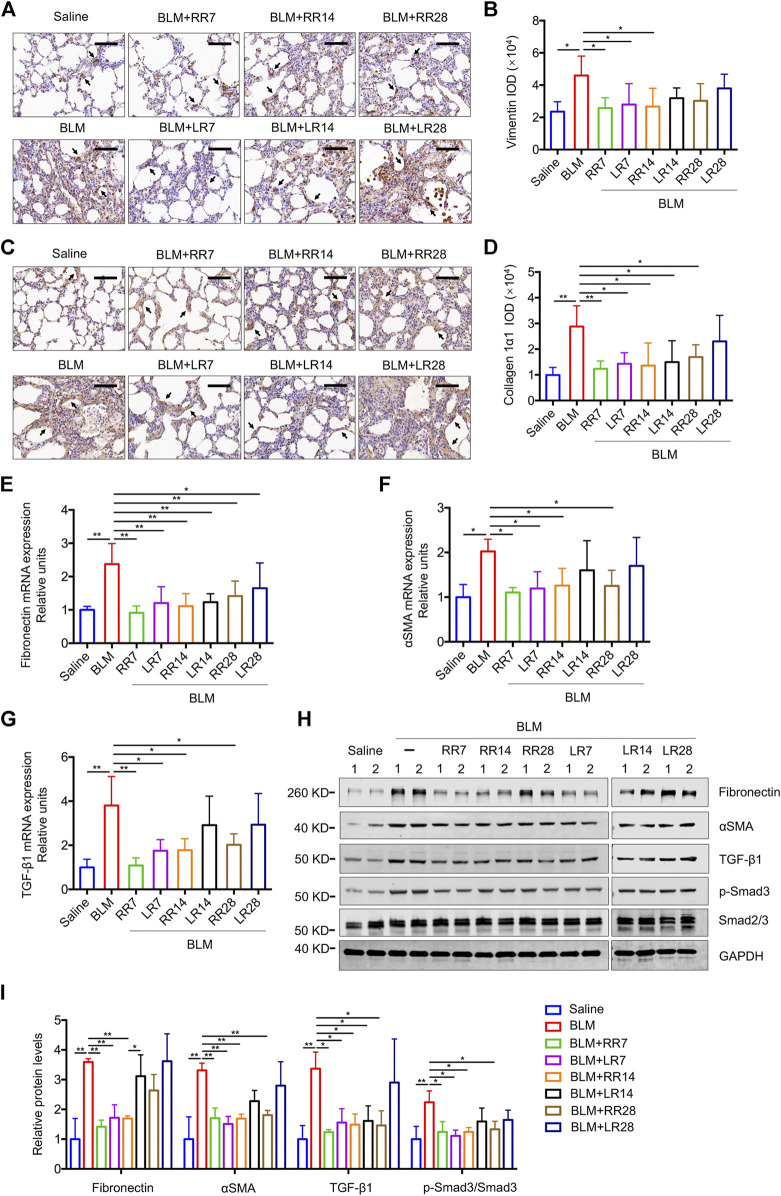
Microcystin-RR (MC-RR) shows a better effect on reducing the expression levels of fibrotic markers than Microcystin-LR (MC-LR). Rats were treated as explained in Figure 3. **(A)** Representative immunohistochemical staining of vimentin in rat lung tissue sections. Scale bar: 100 μm. Black arrows indicate vimentin-positive cells. **(B)** The expression of vimentin was quantified by integrated optical density (IOD) using Image-Pro Plus 6.0 software. **(C)** Representative immunohistochemical staining of collagen 1α1 in rat lung tissues. Scale bar: 100 μm. Black arrows indicate collagen 1α1-positive cells. **(D)** Quantification of collagen 1α1 was performed by IOD. **(E–G)** The mRNA was purified from pulmonary tissues and examined for the expression of fibrotic markers, including fibronectin, α-smooth muscle actin (αSMA) and transforming growth factor-β1 (TGF-β1). **(H,I)** Protein levels in rat lung tissues were measured by western blot and quantified. Data are presented as mean ± SD. *p < 0.05, **p < 0.01 determined by one-way ANOVA with S-N-K post-hoc analysis. Each group had five rats.

We have previously shown that MC-LR exerts a therapeutic effect on BLM-induced rat pulmonary fibrosis and in an FITC-injured mouse model. To evaluate the therapeutic effect of MC-RR, we exposed rats to MC-RR starting on days 7, 14, and 28 after BLM induction corresponding to the inflammation phase, transitional phase of inflammation/fibrosis, and the late stage of fibrosis, respectively, and compared the effects of MC-RR and MC-LR treatment (Figure 3A). Collagen and elastic fibers, the major components of the extracellular matrix, were detected by Sirius Red and EVG staining of the lung tissue. As shown in Figures 3B–E, the deposition of collagen and elastic fibers markedly increased in the lung tissues of BLM model animals as compared with that in the saline controls. Both MC-RR and MC-LR demonstrated obvious therapeutic effects on BLM-induced pulmonary fibrosis. In comparison with MC-LR treatment, MC-RR showed better effects at all three time points, especially in the groups treated at the later stage (RR28 vs. LR28) because the MC-LR group showed a modest reduction in the fiber content. The rats treated with MC-RR had lower Ashcroft scores for the lung tissue, especially those from the RR7 group, suggesting that MC-RR is more effective against fibrosis than MC-LR. MC-RR administration improved the core characteristics of BLM-induced pulmonary fibrosis, as evident from the significant reduction in inflammatory infiltration and interstitial thickening observed only in the early MC-LR intervention groups (LR7 and LR14) (Supplementary Figure S1). We also used immunohistochemical staining to detect the expression of vimentin and collagen 1α1, proteins associated with activated fibroblasts in the lung tissue. The data supported the histopathological observations described above (Figures 4A–D). Furthermore, both MC-RR and MC-LR treatments significantly decreased the mRNA (Figures 4E–G) and protein levels (Figures 4H,I) of αSMA, fibronectin, TGF-β1, and p-Smad3; however, MC-RR exerted a stronger inhibitory effect at most of the observation time points.

### MC-RR Suppresses the M2 Polarization of Macrophages

**FIGURE 5:**
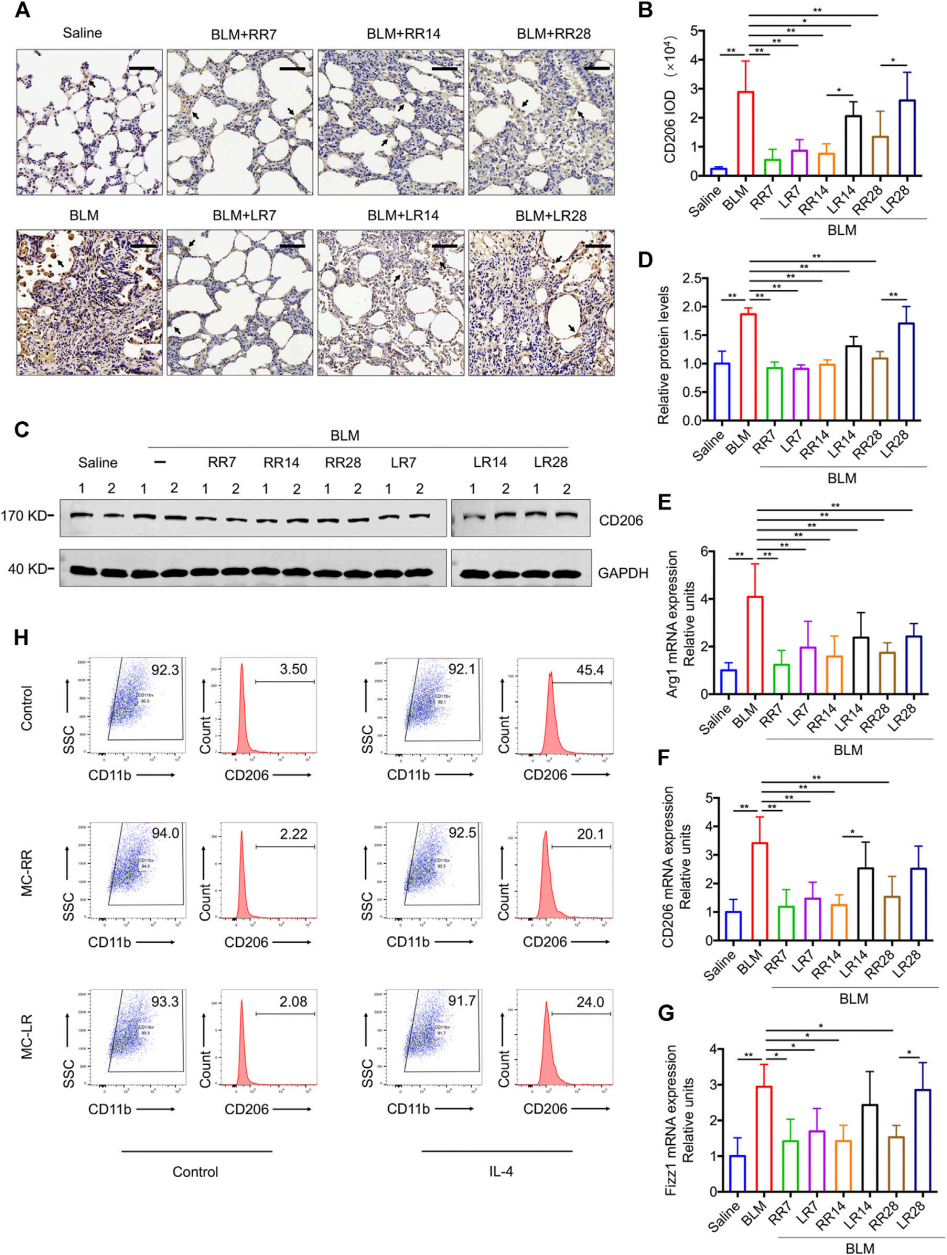
Microcystin-RR (MC-RR) suppresses the expression of M2 macrophage-related markers. **(A–G)** Rats were treated as explained in Figure 3. **(A,B)** Representative immunohistochemical staining and quantification of CD206 in the rat lung tissue sections. Scale bar: 100 μm. Black arrows indicate CD206-positive cells. **(C,D)** The protein level of CD206 in rat lung tissues was measured by western blotting and quantified. **(E–G)** The mRNA expression of arginase-1 (*Arg1*), *CD206*, and resistin-like molecule-α (*Fizz1*) in rat pulmonary tissues was examined by quantitative Reverse-Transcription Quantitative Polymerase Chain Reaction (RT-PCR). Data are presented as mean ± SD. *p < 0.05, **p < 0.01 determined by one-way ANOVA with S-N-K post-hoc analysis. Each group had five rats. **(H)** RAW264.7 cells were left alone or treated with interleukin (IL)-4 (5 ng/ml) for 48 h to induce polarization. In some of the assays, cells were also treated with 0.1 μM MC-RR or MC-LR for the same duration as indicated. Percentages of M2-like macrophages (CD206^+^/CD11b^+^) were analyzed by flow cytometry.

Our previous work showed that MC-LR modulated the M2 macrophage polarization. Here, we investigated whether MC-RR exerts a similar effect on the differentiation of macrophages to the M2 phenotype. In comparison with MC-LR treatment, MC-RR induced a comparable level of iNOS, a key molecular marker for M1 macrophages (Supplementary Figure S2), but had a stronger inhibitory effect on CD206^+^ M2 macrophages and CD206 protein expression (Figures 5A–D). The expression of CD206 was lower in the rats from MC-RR treatment group starting from day 14 (RR14) and 28 (RR28) after BLM exposure than that in their counterparts treated with MC-LR. MC-RR treatment also reversed the mRNA levels of arginase-1 (*Arg1*), *CD206*, and resistin-like molecule-α (*Fizz1*), which were elevated upon BLM exposure (Figures 5E–G).

To evaluate the effect of MC-RR on the macrophage differentiation *in vitro*, we induced M2 polarization in RAW264.7 cells by treatment with IL-4 for 48 h. As expected, IL-4 stimulation expanded the CD206-positive population in cultured RAW264.7 cells from 3.5 to 45.4%. MC-RR treatment reduced the percentage of CD206-positive cells (Figure 5H). To demonstrate whether MC-RR impedes the M2 macrophage-induced EMT and FMT, we established a cell co-culture system consisting of RAW264.7, A549, and NIH3T3 cell lines (Supplementary Figure S3). Upon co-culture with IL-4 pretreated macrophages, A549 cells had decreased E-cadherin and increased vimentin expression, a typical characteristic of mesenchymal cells. Co-cultured NIH3T3 cells showed significantly higher levels of αSMA and fibronectin, which are markers of myofibroblasts. In contrast, MC-RR or MC-LR treatment markedly blocked the above-mentioned EMT and FMT triggered by IL-4-pretreated macrophages. However, no difference was observed between MC-RR and MC-LR treatment groups in the co-culture experiment.

### MC-RR Exhibits Lower Toxicity than MC-LR

**FIGURE 6:**
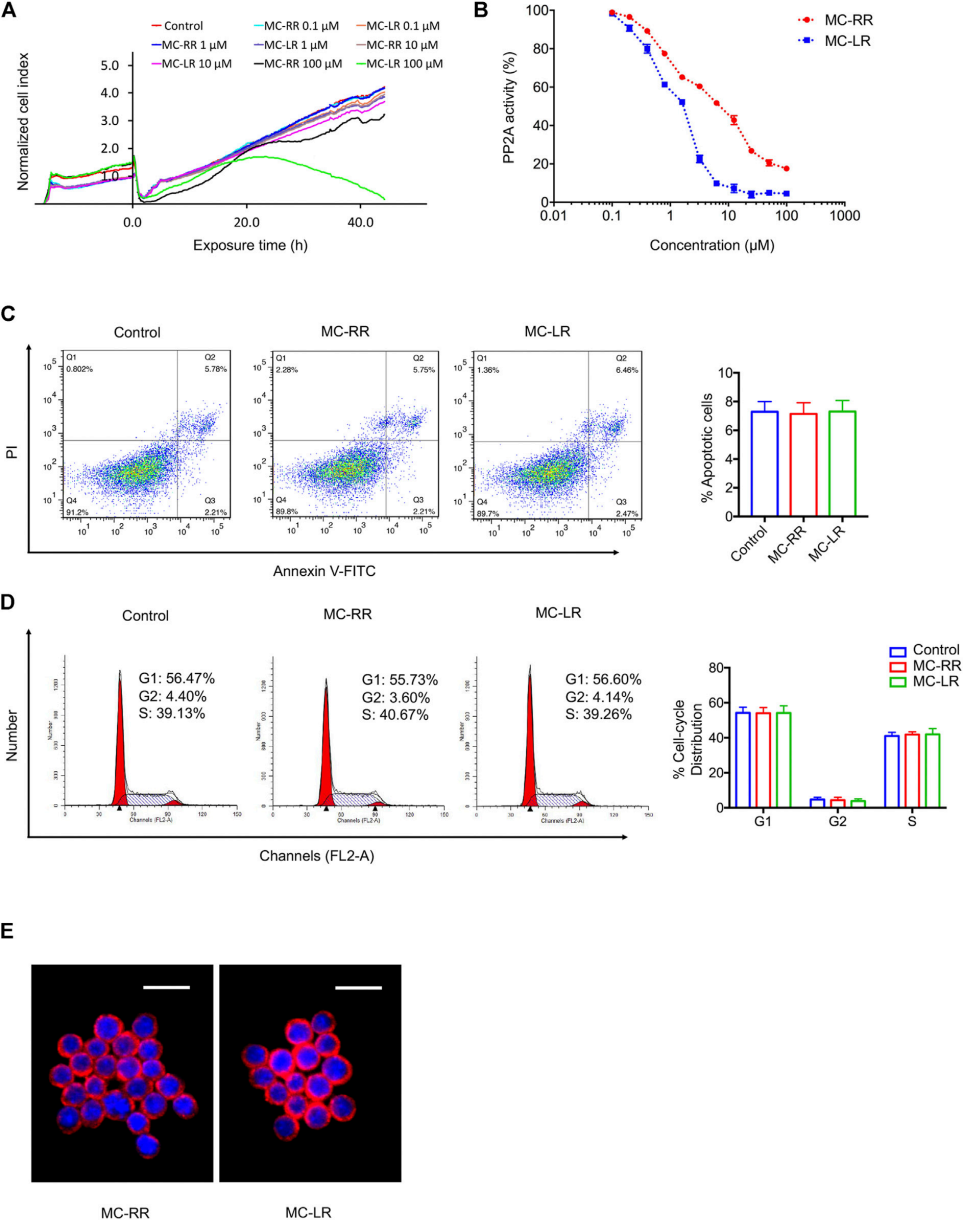
Microcystin-RR (MC-RR) shows lower toxicity than Microcystin-LR (MC-LR). **(A)** Human normal liver cell line L02 susceptible to MCs was exposed to 0, 0.1, 1, 10, and 100 μM MC-RR or MC-LR for 48 h. L02 cell yield was continuously monitored by the xCelligence real-time cell analyzer (RTCA). The cell indices were normalized to reflect the cell growth relative to the values at initial exposure. **(B)** Alterations in protein phosphatases 2A (PP2A) activity were measured in L02 cells exposed to different concentrations of MC-RR or MC-LR. The activity is expressed as the percentage of PP2A activity in extracts from untreated cells. **(C,D)** RAW264.7 cells were cultured with 0.1 μM MC-RR or MC-LR for 48 h. Cell cycle and apoptosis was measured by flow cytometry. Data are presented as mean ± SD. **(E)** The uptake of MC-RR or MC-LR by macrophages was detected by immunofluorescence. DAPI was used for nuclear staining (blue). Scale bar: 20 μm.

To compare the toxicity of MC-RR and MC-LR, we used a human normal liver cell line (L02) that is highly sensitive to MC exposure. The cytotoxicity of MC-RR and MC-LR to L02 cells was monitored using a real-time cell analyzer (RTCA) system (Figure 6A). After continuous incubation with different drug concentrations, the toxicity of MC-RR was significantly lower than that of MC-LR. At low concentrations (≤10 μM), MC-RR and MC-LR did not significantly reduce the cell viability. As the drug concentration was increased to 100 μM, the cell growth curve showed slight fluctuations following MC-RR exposure but presented a rapid decline after MC-LR treatment; no cells survived 48 h after MC-LR exposure. Next, we tested the effect of MC-RR and MC-LR on the inhibition of PP2A activity. Both MC-RR and MC-LR treatments caused a dose-dependent decrease in cellular PP2A activity (Figure 6B). However, the concentration causing a 50% decrease in total PP2A activity was 10 μM for MC-RR and <2 μM for MC-LR. Flow cytometry analysis revealed the absence of any effect on the cell cycle and apoptosis of cultured RAW264.7 cells following 0.1 μM MC-RR treatment, consistent with our previous report on MC-LR (Figures 6C,D). Moreover, there was no difference in cellular uptake of MC-RR and MC-LR in RAW264.7 cells (Figure 6E). We further evaluated the toxicity of MC-RR *in vivo* by determining serum biochemical values and morphological changes in the liver and kidneys of experimental animals (Supplementary Table S2; Supplementary Figure S4). In line with our previous results, the administration of MC-RR or MC-LR did not lead to additional damage to BLM-treated rats. Based on these observations, we conclude that MC-RR is less toxic than MC-LR and, thus, safer for the treatment of pulmonary fibrosis.

## Discussion

IPF is a chronic, progressive, and irreversible fibrosing interstitial pneumonia of unknown cause that occurs mainly in elderly people with limited therapy. Pirfenidone and nintedanib, two FDA-approved antifibrotic drugs, can significantly slow down the decline in the lung function of IPF patients but have not been proven to be effective in disease cessation (Spagnolo et al., 2020a; Spagnolo et al., 2020b). Clinical trials have not yet found a cure for IPF. We have previously reported a novel anti-fibrotic strategy using MC-LR, which effectively inhibited collagen accumulation and ameliorated fibrotic features, especially in the early stage of BLM-associated lung injury. We proved that MC-LR suppressed the TGF-β1 pathway, a key signaling pathway in IPF pathogenesis and differentiation of fibrosis-related M2 macrophages. Given its effect on the reduction in the fibrotic response, we consider that MC-LR could be translated for pulmonary fibrosis therapy. However, attention should be paid to the potential toxicity of MC-LR. In this study, we chose MC-RR, a lower toxic congener of MCs, and evaluated its therapeutic and preventive roles in pulmonary fibrosis.

MCs are a group of cyclic heptapeptide molecules produced by cyanobacteria. More than 100 different congeners of MCs have been identified (Svirčev et al., 2017; Kordasht et al., 2020). Congeners mainly differ in their amino acids at positions 2 and 4, as indicated in their names. MC-LR is one with l-leucine (L) and l-arginine (R) at positions 2 and 4, respectively, while MC-RR presents l-arginine at both positions (Van Apeldoorn et al., 2017). Toxicological studies on experimental mice (LD50) and cultured cells *in vitro* indicated that the toxicity of MC-RR is 1/10 lower than that of MC-LR (Dittmann et al., 2013; Shimizu et al., 2013). We first investigated whether MC-RR exhibits similar anti-fibrotic functions. In the BLM-induced rat model, MC-RR treatment markedly attenuated collagen deposition and inflammation infiltration, the main histological features of pulmonary fibrosis. These data indicate that both MC-RR and MC-LR exert therapeutic effects on pulmonary fibrosis, although their toxicity greatly varies. Thus, the anti-fibrotic effects could be related to the structure of the cyclic heptapeptide shared by these compounds. We have previously reported the anti-fibrotic effect of MC-LR in a therapeutic strategy (Wang et al., 2020). In the present study, we performed a prevention regimen for MC-RR using a BLM-induced mouse model. The mice started to receive MC-RR 7 days prior to BLM intratracheal instillation. Both chest micro-CT scan and histopathological examination of the lung tissue revealed an obvious remission of fibrosis after 4 weeks of BLM induction as compared with that in the control mice, suggesting that MC-RR imparts preventive effects on BLM-induced pulmonary fibrosis.

Next, we compared the effects of low-toxicity MC-RR with high-toxicity MC-LR in the BLM-induced rat model. We found that the administration of MC-RR mirrors the anti-fibrotic effect observed in MC-LR-treated rats, rather exhibits better results. Using Sirius Red and EVG staining, we measured the contents of collagen and elastic fibers, reflective of the deposition of the extracellular matrix mainly produced by myofibroblasts. We also assessed the expression levels of collagen 1α1, fibronectin, αSMA, and vimentin, which are the markers of the main effector cells of pulmonary fibrosis. Our results indicate that MC-RR could significantly ameliorate the features of pulmonary fibrosis even when the intervention was performed at a later stage of BLM-induced fibrogenesis. In line with this result, we detected low TGF-β1 signaling activity in MC-RR-treated lung tissues. In contrast, the rats treated with MC-LR in the later stages of fibrosis development failed to show any significant improvement in the symptoms of pulmonary fibrosis. These results indicate that MC-RR may have a better anti-fibrotic effect, which is probably related to the basic amino acid (l-arginine) at position 2 of the congener.

The alteration in macrophage differentiation in response to specific stimuli and the microenvironment plays a critical role in the pathogenesis of fibrosis (Byrne et al., 2016; Zhang et al., 2018; Aran et al., 2019; Ogger and Byrne, 2020; Rao et al., 2020). M1-type macrophages produce chemokines to recruit inflammatory cells, while M2 macrophages release profibrotic cytokines that contribute to extracellular matrix remodeling. We have previously reported that MC-LR alleviates pulmonary fibrosis by regulating macrophage polarization. To assess the effects of MC-RR on macrophages, we observed the differentiation of macrophages *in vivo* and *in vitro* following MC-RR administration. Our results show that MC-RR reduced the M2 type macrophages in the lung tissues of rats injured by BLM but had no obvious effect on the M1 type cells. In comparison with MC-LR, MC-RR treatment produced a better inhibitory effect on the M2 macrophage differentiation. *In vitro*, MC-RR treatment attenuated the IL-4-stimulated M2 macrophage polarization and suppressed the mesenchymal transition of epithelial cells and myofibroblast differentiation in our co-cultured cell model. These results indicate that MC-RR is a favorable molecule for anti-M2 polarization of macrophages.

Many studies have reported the lower toxicity of MC-RR than that of MC-LR (Ito et al., 2002; Ufelmann et al., 2012; Díez-Quijada et al., 2019; Biales et al., 2020). The acute toxicity test showed that the median lethal dose (LD50, i.p.) in mice was 500–800 μg/kg body weight (b.w.) for MC-RR but 50 μg/kg b.w. for MC-LR (Bouaïcha et al., 2019). Our *in vitro* toxicological analysis confirmed that MC-RR is a lower toxic congener than MC-LR. Further, the LD50 value of MC-LR was 5,000 μg/kg b.w. in mice following oral ingestion, 100 times higher than that by intraperitoneal injection (Fawell et al., 1999; Bouaïcha et al., 2019). The safety of MCs in our experimental program was fully considered. We did not observe any additional damage to the model animals, which further proves that the doses of MC-RR and MC-LR used herein were tolerated by BLM-induced animals. Considering the duration and accumulation of drugs, we believe that MC-RR is safer than MC-LR for the treatment of pulmonary fibrosis.

The toxicity mechanism of MCs has been studied and known to be related to the inhibition of PP1 and PP2A by covalent binding, leading to protein hyperphosphorylation, cytoskeletal modifications, and actin filament disruption. The affinity of MC-LR for PP2A was reported to be 20–24 times higher than that for PP1 (Ito et al., 2002; Meng et al., 2011). Wolf and Frank proposed the use of toxicity equivalent factors (TEFs) for MCs (Wolf and Frank, 2002). The TEFs value was 1.0 for MC-LR and 0.1 for MC-RR. Analysis of the inhibitory potency revealed that MC-RR was a weaker PP2A inhibitor than MC-LR. Likewise, Ufelmann et al. reported that MC-RR was a 50- to 200-fold less potent inhibitor than MC-LR in bovine kidney and human red blood cells (Ufelmann et al., 2012). Trogen et al. demonstrated the similarity in the gross structures of MC-RR and MC-LR, but MC-RR presents a more accentuated and compact saddle structure that may affect the ability to form a tight complex with PP and contribute to the observed toxicity differences between MC-RR and MC-LR (Trogen et al., 1998). However, the differences in the inhibitory potency of MC congeners showed limited coincidence with their cytotoxicity. Hoeger et al. reported that the PP inhibitory capacity does not consistently explain MC toxicity *in vivo* on the basis of the similar IC50 values of MC-RR and MC-LR for PP1 and PP2A (Hoeger et al., 2007). We have demonstrated that MC-RR has a better effect on alleviating pulmonary fibrosis than MC-LR, indicating a novel mechanism other than PP inhibition, which remains to be further explored.

## Data Availability

The original contributions presented in the study are included in the article/Supplementary Material, further inquiries can be directed to the corresponding authors.
