# Supplementary material for: Anti-Fibrotic Effects of Low Toxic Microcystin-RR on Bleomycin-Induced Pulmonary Fibrosis: A Comparison with Microcystin-LR
Source: Front Pharmacol. 2021 Jun 8;12:675907. doi: 10.3389/fphar.2021.675907 (PMC8217630; doi:10.3389/fphar.2021.675907)
Supplement: Supplementary file 1 [file DataSheet1.docx]

**Supplementary Material**

**Supplementary Figure and Figure Legends**

**Supplementary Figure S1 Microcystin-RR (MC-RR) ameliorates bleomycin (BLM)-induced inflammation infiltration.** Rats were treated as explained in Fig. 3. **(A)** Representative H&E staining of rat lung tissue sections. Scale bar: 100 μm. **(B)** Inflammation score of stained sections of rat lung tissues was assessed by two pathologists blinded to study design and is shown as the mean ± SD per group. **P* < 0.05, ***P* < 0.01 determined by one-way ANOVA with S-N-K post-hoc analysis. Each group had five rats.

**
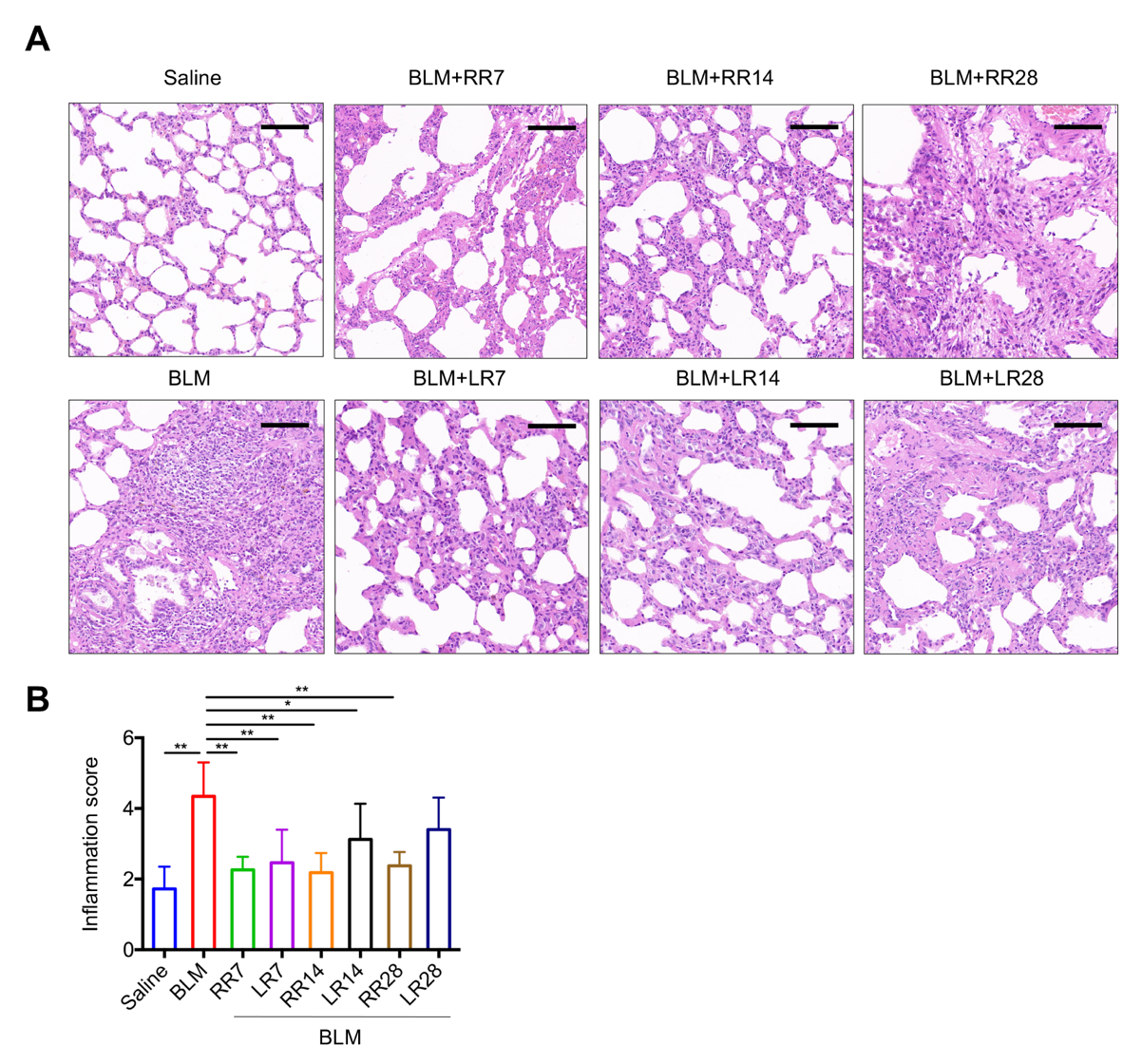
**

**Supplementary Figure S2 Microcystin-RR (MC-RR) does not reduce the expression level of inducible nitric oxide synthase (iNOS)** **in lung tissues of bleomycin (BLM)-induced model rats.** Rats were treated as explained in Fig. 3. **(A)** Pulmonary tissue sections were examined for the expression of iNOS using immunohistochemistry. Scale bars: 100 μm. **(B)** The expression of iNOS was quantified by integrated optical density (IOD) using Image-Pro Plus 6.0 software. Data were presented as mean ± SD. One-way ANOVA with S-N-K post-hoc analysis. Each group had five rats. **(C)** The protein level of iNOS in rat lung tissues was measured by western blot.


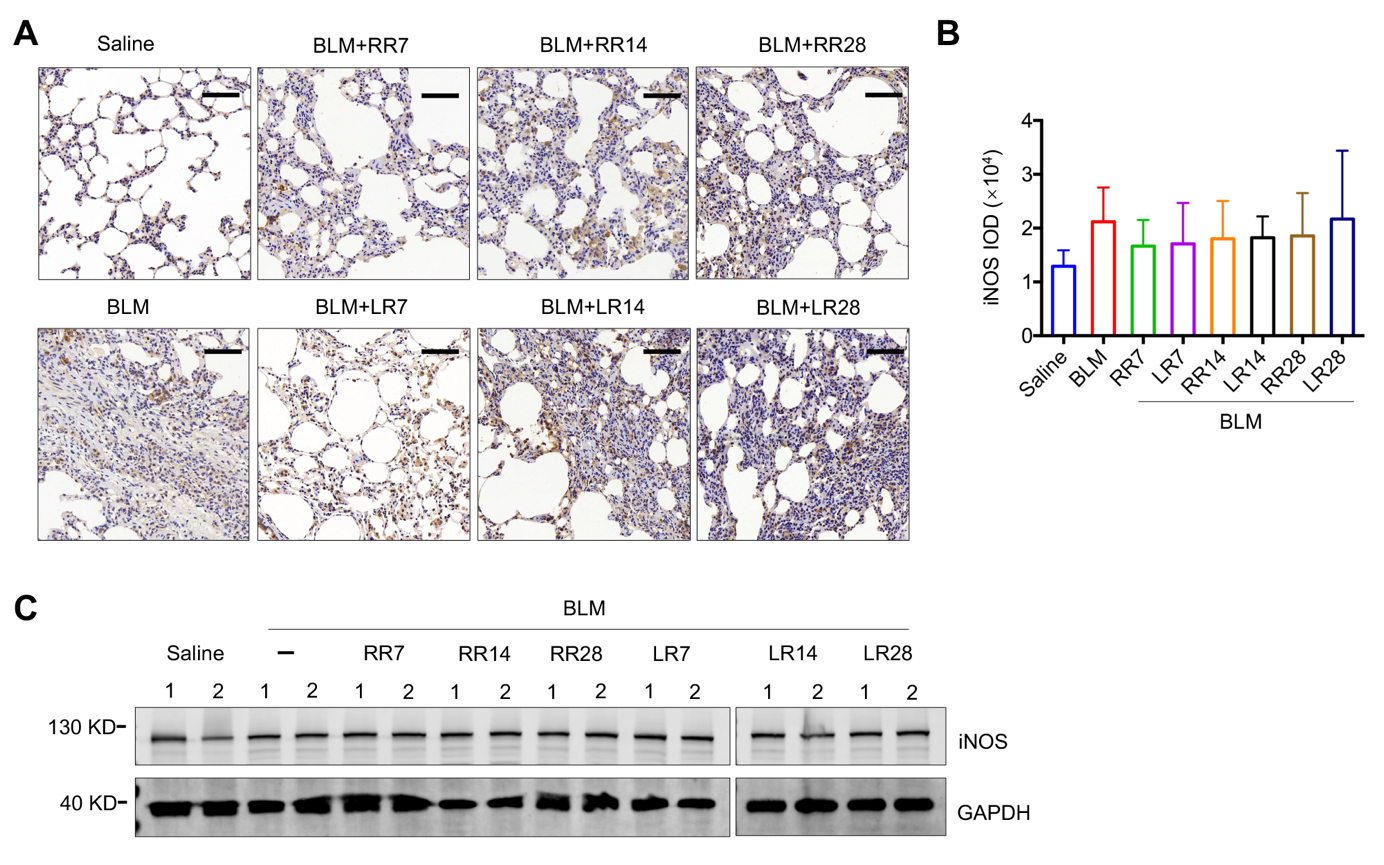


**Supplementary Figure S3 Microcystin-RR (MC-RR) suppresses M2 macrophage-mediated epithelial-mesenchymal transition (EMT) and fibroblast-to-myofibroblast transition (FMT).** A549 (commonly used as a model of human alveolar type II pulmonary epithelium) or NIH3T3 (mouse embryonic fibroblasts) were co-cultured for 48 h with the cell insert containing interleukin (IL)-4 (5 ng/mL) pretreated RAW264.7 cells to induce EMT or FMT. Some of the cells were also treated with 0.1 μM MC-RR or MC-LR as indicated. **(A)** Schematic representation of experimental design. **(B, C)** The expression levels of EMT or FMT markers in A549 and NIH3T3 cells were examined by immunofluorescence staining. Scale bar: 50 μm. **(C, D)** The protein levels of EMT or FMT markers were measured by western blot.


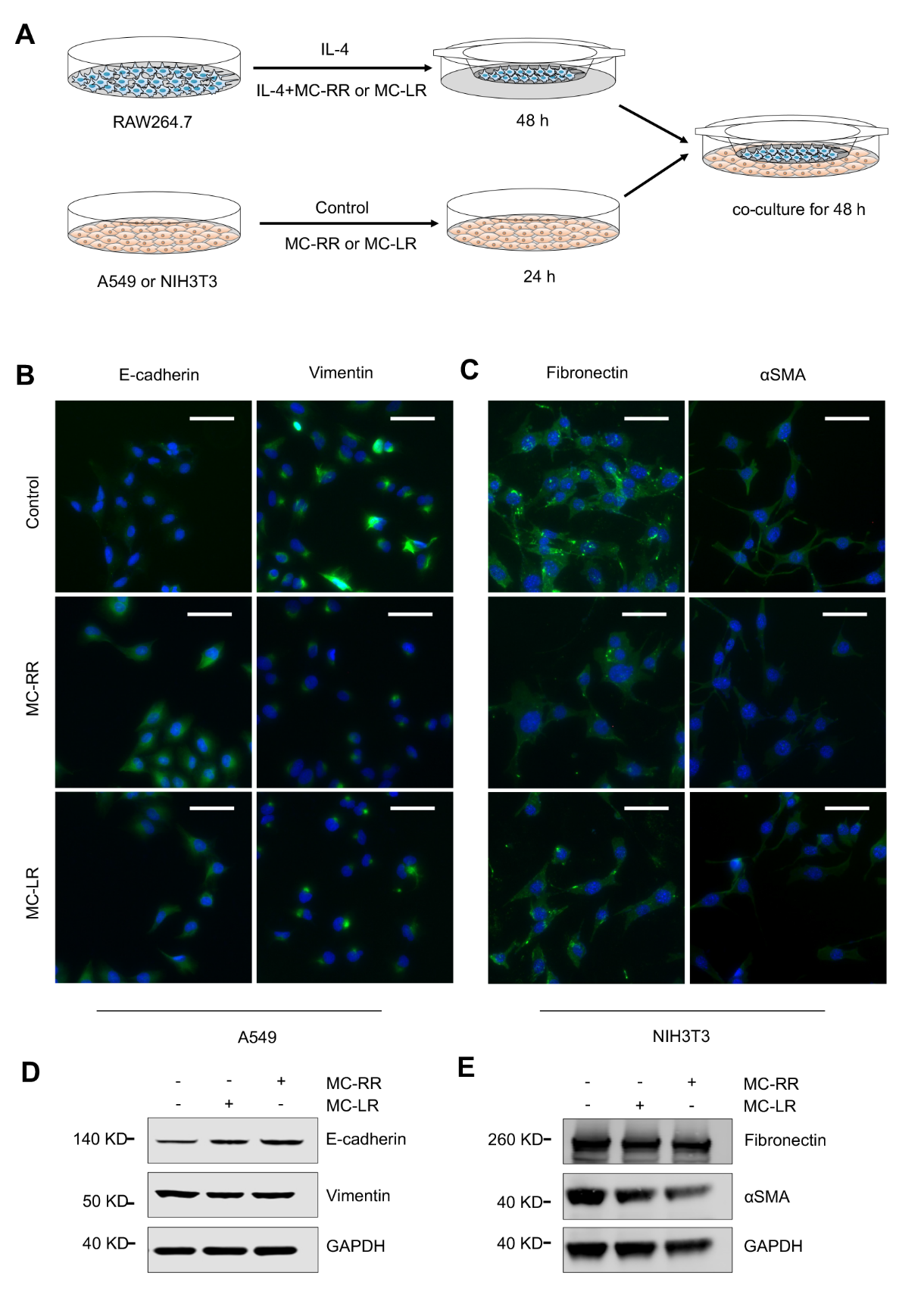


**Supplementary Figure S4 Microcystins (MCs) does not cause obviously histological damage on liver and kidney tissues of the rats with bleomycin (BLM)-induced pulmonary fibrosis.** Rats were treated as explained in Fig.3. Tissue sections of liver and kidneys were prepared and subjected to H&E staining. Scale bar: 200 μm.


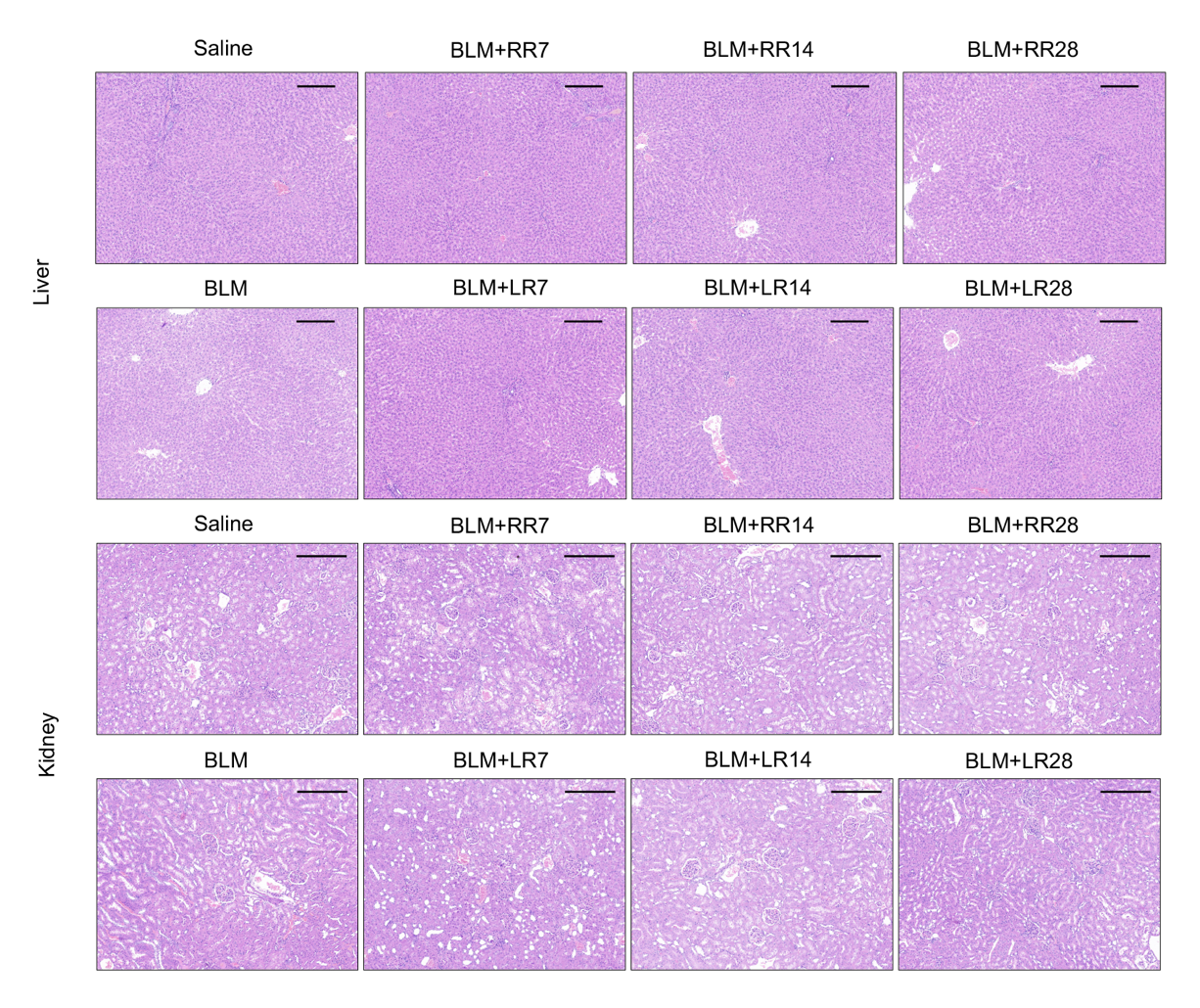


**Supplementary Table S1 Primers for qRT-PCR**

| **Genes** | **Sequences** |
| --- | --- |
| Rat *TGF-β1* | F: AGGGCTACCATGCCAACTTC |
|  | R: CCACGTAGTAGACGATGGGC |
| Rat *GAPDH* | F: AGTGCCAGCCTCGTCTCATA |
|  | R: GGTAACCAGGCGTCCGATAC |
| Rat *αSMA* | F: ACCATCGGGAATGAACGCTT |
|  | R: CTGTCAGCAATGCCTGGGTA |
| Rat *Fibronectin* | F: GGATCCCCTCCCAGAGAAGT |
|  | R: GGGTGTGGAAGGGTAACCAG |
| Rat *CD206* | F: TGATTCCGGTCGCTGTTCAA |
|  | R: GAACGGAGATGGCGCTTAGA |
| Rat *Arg1* | F: TTGGAACGAAACGGGAAGGT |
|  | R: TGTTCGGTTTGCTGTGATGC |
| Rat *Fizz1* | F: CGAGGGGACACTGACTTTCAA |
|  | R: CTGGGACCATCAGCTGGAGA |

**Supplementary Table S2** **Biochemical characteristics measurements of microcystins (MCs) treated rats.**

| **Treatment** | **Saline** | **BLM** | **RR7** | **LR7** | **RR14** | **LR14** | **RR28** | **LR28** |
| --- | --- | --- | --- | --- | --- | --- | --- | --- |
| AST (UI/L)  Mean ± SD | 105.4 ± 15.52 | 107.6 ± 46.72 | 101 ± 10.34 | 107 ± 21.40 | 103 ± 16.46 | 105 ± 21.92 | 103.2 ± 9.99 | 107.2 ± 11.69 |
| ALT (UI/L)  Mean ± SD | 32 ± 7.28 | 38.2 ± 15.83 | 33.8 ± 6.57 | 38 ± 13.29 | 34.2 ± 15.16 | 37 ± 7.28 | 34 ± 11.27 | 36.2 ± 8.7 |
| CRE (mmol/L)  Mean ± SD | 93.6 ± 7.44 | 92.4 ± 7.27 | 94 ± 6.96 | 92.8 ± 18.13 | 95.8 ± 10.47 | 99.4 ± 24.95 | 94.6 ± 10.69 | 98.8 ± 10.11 |
| BUN (mmol/L)  Mean ± SD | 5.62 ± 0.76 | 6.58 ± 1.14 | 5.59 ± 0.64 | 6.19 ± 1.40 | 5.51 ± 0.44 | 6.25 ± 1.59 | 5.69 ± 0.64 | 6.1 ± 0.87 |
| TP (g/L)  Mean ± SD | 52.4 ± 2.97 | 51.8 ± 1.78 | 51.4 ± 5.08 | 55.4 ± 4.10 | 53.4 ± 0.55 | 54 ± 3.74 | 51.8 ± 4.66 | 52.4 ± 1.14 |
| ALB (g/L)  Mean ± SD | 30.2 ± 0.84 | 30.6 ± 0.55 | 30.4 ± 4.72 | 31.8 ± 1.64 | 31.2 ± 0.84 | 31.8 ± 0.84 | 30.6 ± 3.21 | 32 ± 5.79 |
| TG (mmol/L)  Mean ± SD | 1.47 ± 0.30 | 1.53 ± 0.67 | 1.48 ± 0.34 | 1.48 ± 0.44 | 1.52 ± 0.18 | 1.50 ± 0.38 | 1.52 ± 0.25 | 1.49 ± 0.38 |
| TC (mmol/L)  Mean ± SD | 1.90 ± 0.33 | 1.94 ± 0.20 | 1.91 ± 0.15 | 1.93 ± 0.16 | 1.92 ± 0.17 | 1.92 ± 0.35 | 1.92 ± 0.19 | 1.92 ± 0.19 |
| HDL (mmol/L)  Mean ± SD | 1.26 ± 0.18 | 1.25 ± 0.82 | 1.27 ± 0.32 | 1.26 ± 0.18 | 1.25 ± 0.12 | 1.26 ± 0.19 | 1.24 ± 0.27 | 1.26 ± 0.15 |
| LDL (mmol/L)  Mean ± SD | 1.57 ± 0.28 | 1.56 ± 0.14 | 1.56 ± 0.34 | 1.57 ± 0.17 | 1.57 ± 0.32 | 1.57 ± 0.28 | 1.58 ± 0.41 | 1.56 ± 0.19 |

Rats were treated as explained in Fig. 3. The serum was separated from whole blood collected from abdominal aorta and examined for the biochemical parameters as indicated. Data was analyzed by one-way ANOVA with S-N-K post-hoc analysis. RR, MC-RR; LR, MC-LR; AST, aspartate aminotransferase; ALT, alanine aminotransferase; CRE, creatinine; BUN, blood urea nitrogen; TP, total protein; ALB, albumin; TG, triglycerides; TC, cholesterol; HDL, high density lipoprotein; LDL, low density lipoprotein. n = 5
